# Understanding the impacts of child marriage on the health and well-being of adolescent girls and young women residing in urban areas in Egypt

**DOI:** 10.1186/s12978-021-01315-4

**Published:** 2022-01-15

**Authors:** Shatha Elnakib, May Elsallab, Maha Abdel Wanis, Shadia Elshiwy, Nishan Prasana Krishnapalan, Nada Aghar Naja

**Affiliations:** 1 Johns Hopkins Bloomberg School of Public Health, Maryland, USA; 2 UNFPA, Cairo, Egypt; 3 UNFPA Arab States Regional Office, Cairo, Egypt

**Keywords:** Child marriage, Adolescent health, Women, Sexual and reproductive health, Egypt

## Abstract

**Background:**

Egypt has made progress in delaying age at marriage, but child marriage continues to be practiced in many places across the country. This study investigates the impacts of child marriage on the health and wellbeing of girls residing in urban Egypt using a multi-method approach.

**Methods:**

The quantitative component leveraged data from the 2014 Egypt Demographic and Health Survey and focused on (1) reproductive health, (2) maternal health and (3) social outcomes among a subsample of ever-married urban women ages 20–24 (N = 1041). Simple and multivariable logistic regressions were used to estimate prevalence odds ratios and 95% confidence intervals for associations between child marriage and the three sets of outcomes. The qualitative component drew from 11 focus groups, 23 in-depth interviews, and 13 key informant interviews conducted in three urban sites in Egypt. The data was thematically analyzed using a combination of inductive and deductive coding.

**Results:**

The prevalence of marriage under age 18 was 13.22%. Child marriage was significantly associated with ever use of contraception (Adjusted Odds Ratio (AOR) 2.95 95% CI 1.67–5.19), multiple births (AOR 12.93 95% CI 5.45–30.72), rapid repeat childbirth (AOR 2.20 95% CI 1.34–3.63), and pregnancy termination (AOR 1.89 95% CI 1.11–3.23). Many of these associations disappeared after adjusting for marriage duration. Girls married under age 18 had larger spousal age gaps (AOR 2.06; 95% CI 1.24–3.41) and higher odds of FGM (AOR 2.14; 95% CI 1.11–4.13). They were significantly more likely to report receiving no ANC care (AOR 0.39; 95% CI 0.19–0.80), and less likely to deliver through C-section (AOR: 0.53; 95% CI 0.34–0.83). Consequences emerging from the qualitative data centered around five themes: (1) Access to and use of sexual and reproductive health services; (2) exposure to FGM; (3) marriage and birth registration; (4) marital relations; and (5) relationship with in-laws.

**Conclusion:**

Findings provide important insights into the practice of child marriage in urban areas in Egypt and illustrate a range of adverse consequences associated with the practice.

## Plain language summary

Child marriage has been shown to lead to a host of negative health and social consequences. In this study, we explored the prevalence and consequences of child marriage in urban areas in Egypt, where there has been little research done on the topic. We analyzed quantitative data collected as part of the Egypt Demographic and Health survey as well as qualitative data collected as part of a bigger study on child marriage in humanitarian studies. In the quantitative analysis, we focused on three sets of outcomes: reproductive health outcomes, social outcomes and maternal health outcomes. In the qualitative analysis, we analyzed focus group discussions, in-depth interviews and key informant interviews and came up with themes around consequences of child marriage. The analysis revealed that child marriage is associated with multiple adverse consequences. However, many of the reproductive health consequences identified were due to the longer duration of these marriages, and thus disappeared when marriage duration was taken into account. From the social outcomes, we found that child marriage was associated with female genital mutilation and larger spousal age gaps, and from the maternal health outcomes, we found that child marriage was associated with not receiving antenatal care and not delivering via C-section. The qualitative data highlighted a range of consequences focusing on access to sexual and reproductive health services, exposure to FGM, marriage and birth registration, and relationships with husbands and in-laws.

## Background

Over the past two decades, Egypt has made substantial progress in delaying age at marriage [1]. However, child marriage, defined as marriage or informal union below the age of 18, continues to be common, with 17.4% of women aged 20–24 marrying before the age of 18 and 2% marrying before age 15 [2]. These rates vary across the country, with rural areas faring generally worse than urban areas [2].

Child marriage is increasingly viewed as a violation of human rights and as a major development issue. The urgency to end child marriage is reflected in the Sustainable Development Goal target 5.3 which aims to ‘eliminate all harmful practices, such as child, early and forced marriage and female genital mutilations.’ Child marriage has been linked with a set of health, economic and social impacts, which have been confirmed in various settings [3]. Child brides are more likely to experience unintended pregnancies and high fertility, to report never using contraceptive methods, and to have reduced access to general health services and specifically maternal healthcare [3–6].They are often in relationships characterized by large age differentials and as a result may have reduced bargaining power and decision-making [7–10]. Their risk of experiencing intimate partner violence is elevated compared to their counterparts who marry at later ages [7]. In come contexts, child marriage is directly or indirectly associated with female genital mutilation (FGM), with both practices sharing many of the same drivers and FGM considered as a prerequisite for marriage in many places [11]. Further, by interrupting or altogether ending girls’ education, child marriage has long-lasting consequences on expected earnings and household welfare, with documented impacts on household consumption and food adequacy [12].

In Egypt, the 2008 Child Law prohibits marriage of girls under age 18 by banning registration of marriage contracts if any of the parties is below the legal age of marriage [13]. However, the current legal system falls short of criminalizing perpetrators of child marriage and as a result, girls continue to get married below age 18 through customary or religious marriages which are not registered with the state [13]. Over the past decade, there have been several national strategies and actions to eliminate violence against children, including child marriage, and in 2014, the National Population Council (NPC)—the entity in charge of developing population policies nationally—launched a five-year national strategy to prevent child marriage. However, political upheaval and changes in government slowed down implementation [13, 14].

A confluence of factors converges to increase girls’ risk of child marriage in Egypt. A recent analysis examining political and socioeconomic factors that drive child marriage in six countries in the Middle East and North Africa (MENA) revealed that discriminatory gender norms and practices, undergirded by religious and cultural beliefs, combined with reduced agency and voice of girls, fear of sexual violence as well as barriers to education are salient drivers of child marriage in this context [14]. Other studies have found that girls belonging to lower socioeconomic classes, and with lower education attainment and a lower levels of literacy are at increased risk of being married as children [1, 13].

While the adverse consequences of child marriage have been explored and documented in other settings, there is a dearth of empirical research on the consequences of child marriage for women and girls in Egypt, and few studies have examined the impacts of this practice in urban contexts in the country. This lack of evidence impedes the design of policies and programs that can effectively address this harmful practice.

Our study draws on quantitative and qualitative data to explore the impacts of child marriage on the health and wellbeing of adolescent girls and young women residing in urban areas in Egypt. The quantitative component of this study leverages existing data from the 2014 Egypt Demographic and Health Survey (EDHS); while the qualitative component uses data collected between February 2019 and August 2019 as part of a broader project that examines child marriage among Syrian refugees and Egyptians.

## Methods

This study employs a multi-method approach that uses quantitative and qualitative data to understand the impacts of child marriage on the health and social wellbeing of girls residing in urban settings in Egypt. We used data from Egypt’s 2014 DHS to examine consequences of child marriage using a sample of ever-married urban women ages 20–24^1^. Additionally, we collected qualitative data using in-depth and key informant interviews and focus group discussions to provide a textured and nuanced analysis of the current experiences of child brides—defined as girls aged 10 to 19 married under age 18—in three urban sites.

### Quantitative component

#### Study setting and data source

We examine child marriage in urban settings in Egypt given that the preponderance of existing research on child marriage has exclusively focused on rural areas in Upper Egypt [18–21]. As such, our quantitative analysis was restricted to a sample of ever-married women ages 20–24 residing in urban areas in Egypt who were interviewed as part of the 2014 EDHS (N = 1041). EDHS is the largest population-based survey conducted in Egypt under the umbrella of the Ministry of Health and Population (MOHP) and implemented by El-Zanaty and Associates [2]. It collects data from women of reproductive age (15–49) on household composition, age at marriage, birth history, socioeconomics, reproductive and maternal health outcomes and FGM among other data. The 2014 survey employed a four-stage stratified cluster design in which each of Egypt’s 27 governorates was first stratified into urban and rural areas, yielding a total of 51 sampling strata (some governorates were exclusively urban). In the first sampling stage, 926 shiakhas/villages were selected as the primary sampling units (PSUs) but were reduced to 884 PSUs due to the exclusion of clusters in Sinai for security reasons. Households were selected in the fourth and final stage, and there were around 15 households sampled per segment. All ever-married women aged 15–49 who resided in the selected households were eligible to be interviewed.

#### Dependent variables

We examined three sets of dependent variables: (1) reproductive health outcomes; (2) social outcomes; and (3) maternal health outcomes that were available in DHS. Reproductive health outcomes included *ever use of contraception, whether childbirth occurred in the first year of marriage, knowledge of fertile period, multiple births (3* +*), rapid repeat childbirth, having at least one unwanted pregnancy, and pregnancy termination*. Ever use of contraception was measured by asking participants whether they ever (in their lifetime) used any *method (modern, traditional, or folkloric) to* delay or avoid getting pregnant and was coded as 1/0. Whether childbirth occurred in the first year was coded 1/0 based on an item that measures the interval between respondents’ first marriage and first birth in months and was restricted to those who already initiated childbirth. Knowledge of fertile period was assessed by a question in which participants were asked when in the ovulatory cycle a woman is most likely to become pregnant if she has sexual relations. Those who responded that a woman is most fertile in the middle of the cycle were coded as 1 and all other responses were coded as 0. Participants who reported having 3 or more births were classified as having multiple births. Rapid repeat childbirth was measured via single items measuring the time that elapsed from each birth and the birth preceding it. Rapid repeat childbirth was defined as having had a birth less than 24 months after a previous childbirth [5]. Those who had no history of childbirth were excluded from this analysis; while those who reported a birth less than 24 months from a previous childbirth were coded as 1. Having at least one unwanted pregnancy was determined based on questions on whether each child was wanted at the time, wanted later, or not wanted at all. Those who stated that one or more of their children was wanted later or not at all were classified as having had at least one unwanted pregnancy and coded as 1. Pregnancy termination was assessed by asking respondents whether they ever (in their lifetime) had a pregnancy that terminated in a miscarriage, abortion, or still birth. Those who reported ever having terminated a pregnancy were coded as 1. All others were coded as 0.

Social outcomes included *polygamy, spousal age gap, and FGM*. Polygamy was assessed by asking participants whether they were in a polygamous union. Spousal age gap was measured by asking participants about the age of their spouse and calculating the difference between respondent’s age and that of her husband. This was then categorized into an age gap of ten or more years which was coded as 1, and an age gap of less than 10 years which was coded as 0. FGM was measured through asking respondents whether they were circumcised; those who responded affirmatively were coded 1.

Maternal health outcomes included any *antenatal care (ANC) visits, institutional delivery, delivery by C-section, and having a child with low birth weight.* For simplification, these were restricted to last births only. ANC visits were coded as 1, defined as attending at least one ANC visit during pregnancy and 0, defined as no ANC visits. Institutional delivery was defined as delivery in public or private health facilities and was coded as 0 if delivery took place at home. Delivery by C-section was defined as 1 if the participant responded undergoing a C-section and 0 otherwise. Having a child with low birth weight was assessed by asking participants to recall the weight of their last child at birth or was recorded from a health card. Participants reporting a birth weight less than 2499 g were coded 1 (low birth weight) and those reporting a birth weight greater than or equal to 2500 g were coded 0.

#### Independent variables

The main independent variable was child marriage, defined as marriage or cohabitation before the age of 18. Participants who reported age of marriage below 18 were classified as having experienced child marriage. We additionally adjusted for the following variables: a DHS-created household wealth index based on household assets which was classified into tertiles instead of quintiles to avoid sparse categories; highest level of education, which was classified into four categories (none, incomplete/complete primary, incomplete/complete secondary, and higher); and marriage duration which was modelled as a continuous variable.

#### Data analysis

We drew from the analytical approach used by Raj et al. [5]. We first calculated the prevalence of child marriage in a sample of urban women aged 20–24 years and presented descriptive statistics using weighted and unweighted percentages. To generate estimates of child marriage based on a denominator of *all women* instead of *ever-married women*, we used an “all women factor” as recommended by the DHS guide [22]. We assessed associations between child marriage and the three sets of dependent variables using single and multivariable logistic regressions which generated prevalence odds ratios (OR) and corresponding 95% confidence intervals (CI). We controlled for wealth, education and age, but age was removed due to its collinearity with education. For some variables which are likely to be affected by lengthier marriage duration as a result of child marriage, we adjusted for marriage duration in addition to the aforementioned sociodemographic variables.

We accounted for DHS survey features as well as selection probability, nonresponse, and sampling differences by using the ‘svy’ function in Stata version 16 as recommended by the DHS guidance [22].

### Qualitative component

#### Study sites and sample

Qualitative data collection took place in three purposively selected urban sites in Egypt: urban Giza, urban Damietta, and urban Qalyubia. The sites have varying levels of child marriage: 21.82% in urban Giza, 16.24% in urban Damietta and 11.48% in urban Qalyubia [2]. In this analysis, we draw from (1) 11 focus group discussions (FGDs)—with participative ranking methodology and photo-elicitation conducted with 86 married and unmarried adolescent girls—defined as girls aged 10–19, and mothers and fathers of adolescent girls; (2) 23 in-depth interviews (IDIs) conducted with married and unmarried girls as well as mothers of adolescent girls; and (3) 13 semi-structured key informant interviews (KIIs) conducted with health providers, community leaders and a legal expert (Table 1).

**Table 1:** Data collection method and number of participants

Data collection method	Type of participant	Total
In -depth interviews	Married girls	4
	Unmarried girls	10
	Mothers	9
	Total	23
Focus group discussions	Married girls	12
	Unmarried girls	28
	Mothers	24
	Fathers	22
	Total	86
Key informant interviews	Community Leader	6
	Legal expert	1
	Health provider	6
	Total	13
Total		122

#### Data collection

Data collection took place between February 2019 and August 2019. Five experienced Egyptian data collectors were recruited and trained in a six-day in-depth training. The training included modules on the research protocol, human subjects research, qualitative interviewing, critical and epistemological reflexivity, and participatory research. This was followed by a refresher training in April.

Participants in the study were identified from UNFPA’s and UNICEF’s beneficiary databases. UNFPA and UNICEF support several Safe Spaces and Family Centers in each of the three governorates. As a starting point, we recruited new users of UNFPA-and UNICEF- supported services, and who fulfilled preset eligibility criteria, then used snowball sampling to identify additional participants. Participants were contacted by phone or in-person by UNFPA Safe Spaces / UNICEF Family Centers staff and were read a recruitment script to confirm their eligibility. To be eligible, participants had to belong to one of the groups mentioned above and had to have lived in the governorate at least one month in the last year. Key informants were recommended by UNICEF, UNFPA and their local partners—Terre Des Hommes, Ministry of Youth and Sports, and Etijah NGO. Second-wave participants were then selected via snowballing.

The FGDs, IDIs and KIIs with community leaders took place in UNFPA-supported Safe Spaces and UNICEF-supported Family Centers. The key informant interview with the legal expert took place remotely (via Skype), and the interviews with health providers took place in health centers/clinics. One facilitator and one note-taker conducted the FGDs and one data collector conducted the IDIS and KIIs. The interviewer was always of the same gender as the participant. Notetakers were asked to take thorough notes documenting verbal and non-verbal communication and to collect participants’ sociodemographic data at the outset of the interview/FGD. Facilitators were tasked with taking informed consent and moderating the discussion. Data collectors used discussion guides which were prepared in collaboration with UNFPA and UNICEF staff around key topics of programmatic relevance to the team. The discussion guides underwent an extensive review process with the local data collection team to ensure that questions were culturally appropriate and adequately phrased. All tools were additionally piloted and subsequently reviewed once more.

#### Data analysis

All FGDs and interviews were audio-recorded and subsequently transcribed verbatim into Arabic. The first author cross-referenced a random sample of transcripts with the audio-recordings to ensure that the transcription was accurate. To ensure proper discussion of challenges and initial findings, and to backstop emerging problems, frequent debriefings took place with the data collection team. Data collectors were encouraged to keep copious fieldnotes and to provide thick descriptions of their surroundings. Notes from the debriefing as well as field notes were amassed and analyzed alongside the transcripts.

In collaboration with the local team, a codebook was developed using deductive codes and was expanded using an inductive coding process in which initial coding was done (line-by-line analysis and incident-to-incident analysis), followed by focused coding in which initial codes were consolidated into focused codes [23] These were then used to sift through the remaining transcripts. Detailed memos were used to draw connections in the data and make comparisons across participant narratives. Dedoose v 8.2.27 [41] was used to facilitate data management and organization. An analysis workshop was organized in August in which preliminary findings were reviewed with the in-country data collection team as well as local partners. The workshop enhanced the validity and credibility of the findings by ensuring that interpretations of the data and the conclusions that were drawn were accurate and unbiased. It also allowed additional reflection on study findings and implications for programmes and policies.

#### Ethics and informed consent

Ethical approval for the research was granted by the Johns Hopkins Bloomberg School of Public Health Institutional Review Board (IRB) [Ref number 9276] and the Egyptian Society for Healthcare Development Research Ethics Committee [Ref number 1/2019]. We sought permission to obtain verbal consent from adult participants and married girls ages 16–17 who are considered emancipated minors in this setting. Oral assent coupled with permission from a parent/guardian was obtained from unmarried children 10–17 and married children under age 16.

## Results

### Quantitative results

#### Sample characteristics

We analyzed a sample of 1,041 ever-married women residing in urban areas in Egypt. Characteristics of the study sample are displayed in Table 2. The prevalence of marriage under age 15 was 2.98% and the weighted estimate which accounts for all women was 2.54%. Marriage under age 18 was more common in the sample, with 20.17% of ever-married women reporting an age of marriage below age 18. The weighted estimate which accounts for all women reveals that child marriage is practiced by a little over one tenth of women (13.22%). The majority of women had a high school education or higher (85.6%) and belonged to the highest wealth tertile (89.2%). The majority of respondents were Muslim, and only 5.7% were Christian.

**Table 2:** Prevalence of child marriage and sample characteristics of girls aged 20–24 residing in urban areas in Egypt

	Number of participants (N = 1041)	%	Weighted %
Age of marriage (years)			
< 15	31	2.98	2.54^**+**^
< 18	210	20.17	13.22^**+**^
Age of women (years)			
20	125	12.01	9.88
21	132	12.68	13.44
22	231	22.19	21.99
23	259	24.88	24.25
24	294	28.24	30.45
Highest level of education			
None	74	7.11	7.74
Primary	73	7.01	6.69
Secondary	695	66.76	66.44
Higher	199	19.12	19.13
Wealth categories			
Poorest/poor	124	11.91	6.88
Middle	56	5.38	3.9
Richer/richest	861	82.71	89.23
Religion			
Muslim	986	94.72	94.28
Christian	55	5.28	5.72
Marriage duration (years)			
Less than 5	810	77.81	75.1
5–12	231	22.19	24.94

^+^Adjusted by the all-women factor to generate an of child marriage based on a denominator of all women instead of ever-married women only

### Child marriage and selected health and social outcomes

Around 18.6% of girls married under age 18 reported having never used contraception, and 45.2% had given birth in their first year of marriage. A quarter reported having three or more childbirths. Around one tenth of girls had at least one unwanted pregnancy and 18.10% terminated a pregnancy. Only 5 girls reported being in polygamous relationships, and 31.4% were in age-disparate relationships with men who are 10 or more years older. FGM was commonly practiced among this group, with 77.14% reporting having undergone FGM. In terms of maternal health outcomes, around 16% of girls married under age 18 reported receiving no antenatal care, and 13% reported that their last child was delivered at home. Delivery by C-section was not uncommon in this group, with 43% of girls reporting C-section as the mode of delivery for their last child. Around 22% reported having a child with low birth weight.

### Association between child marriage and selected health and social outcomes

Table 3 illustrates results of the simple and multivariable regression analysis. Simple regression analysis revealed that women married as children compared to those married as adults were significantly less likely to correctly identify a woman’s fertile period (OR 0.6; 95% CI 0.38–0.96), more likely to report multiple births (OR 19.1 95% CI 8.03–45.39), to experience rapid repeat pregnancy (OR 2.65 95% CI 1.60–4.40) and to terminate a pregnancy (OR 2.02; 95% CI 1.20–3.39). Women married as children were also more likely to report ever use of contraception (OR 2.69 95% CI 1.62–4.44). In terms of social consequences, they were more likely to be in age-disparate relationships (OR 2.22; 95% CI 1.35–3.63) and more likely to have undergone FGM (OR 3.33; 95% CI 1.77–6.27). They were additionally at higher risk of experiencing adverse maternal health outcomes. Compared to their counterparts who married later, girls married under age 18 were less likely to receive any antenatal care (OR 0.27; 95% CI 0.13–0.56), less likely to have institutional deliveries (OR 0.31; 95% CI 0.15–0.65), more likely to deliver babies with low birth weight (OR 2.00; 95% CI 1.03–3.90). They were also less likely to deliver via C-section (OR 0.43; 95% CI 0.28–0.65).

**Table 3:** Crude and adjusted associations between child marriage and several fertility and empowerment-related outcomes

Outcome Variable	Married < 18 (N = 210)		Married ≥ 18 (N = 831)		OR (95% CI)	AOR (95% CI)	AOR (95% CI)^$^
	**N**	**%**^**a**^	**N**	**%**^**a**^			
Reproductive health outcomes							
Ever use of contraception							
Yes	171	81.43	498	59.93	2.69 (1.62–4.44)*	2.95 (1.67–5.19)*	0.26 (0.14 –0.47)*
No	39	18.57	333	40.07	Reference	Reference	Reference
Childbirth in the first year							
Yes	89	45.18	362	59.93	0.72 (0.47–1.10)	0.82 (0.52–1.28)	NA
No	108	54.82	242	40.07	Reference	Reference	Reference
Knowledge of ovulation period							
Yes	50	23.81	301	26.22	0.6 (0.38–0.96)*	0.87 (0.53–1.43)	1.03 (0.54–1.95)
No	160	76.19	530	63.78	Reference	Reference	Reference
Multiple births (3 +)							
Yes	48	22.86	15	1.81	19.1 (8.03–45.39)*	12.93 (5.45–30.72)*	1.59 (0.37–6.79)
No	162	77.14	816	98.19	Reference	Reference	Reference
Rapid repeat childbirth							
Yes	64	32.49	96	15.89	2.65 (1.60–4.40)*	2.20 ( 1.34–3.63)*	0.90 (0.42–1.92)
No	133	67.51	508	84.11	Reference	Reference	Reference
At least one unwanted pregnancy							
Yes	19	10.11	64	10.60	1.07 (0.54–2.16)	1.04 (0.50–2.19)	0.57 (.22–1.48)
No	169	89.89	540	89.40	Reference	Reference	Reference
Pregnancy termination							
Yes	38	18.10	97	11.67	2.02 (1.20–3.39)*	1.89 (1.11–3.23)*	0.97 (0.40–2.34)
No	172	81.90	734	88.33	Reference	Reference	Reference
Social outcomes							
Polygamy							
Yes	5	2.39	13	1.56	0.69 (0.13–3.72)	0.39 (0.07–2.25)	NA
No	204	97.61	818	98.44	Reference	Reference	
Spousal age gap							
Yes	66	31.43	145	17.45	2.22 (1.35–3.63)*	2.06 (1.24–3.41)*	NA
No	144	68.57	686	82.55	Reference	Reference	
FGM							
Yes	162	77.14	578	69.64	3.33 ( 1.77–6.27)*	2.14 (1.11–4.13)*	NA
No	48	22.86	252	30.36	Reference	Reference	
Maternal Health outcomes							
Any ANC visits (last birth)							
Yes	158	84.49	575	95.36	0.27 (0.13–0.56)*	0.39 (0.19–0.80)*	NA
No	29	15.51	28	4.64	Reference	Reference	
Institutional delivery (last birth)							
Yes	162	86.63	569	94.36	0.31 (0.15–0.65)*	0.49 (0.23–1.02)	NA
No	25	13.37	34	5.64	Reference	Reference	
Delivery by C-section (last birth)							
Yes	80	42.78	387	64.18	0.43 (0.28–0.65)*	0.53 (0.34–0.83)*	NA
No	107	57.22	216	35.82	Reference	Reference	
Low birth weight (last birth)							
Yes	23	21.70	56	13.53	2.00 (1.03–3.90)*	1.62 (0.77–3.40)	NA
No	83	78.30	358	86.47	Reference	Reference	

^a^Unweighted estimates

*p value < 0.05

^$^Additionally adjusted for duration of marriage

After adjusting for girls’ education and wealth, child marriage continued to be significantly associated with ever use of contraception (Adjusted Odds Ratio (AOR) 2.95 95% CI 1.67–5.19), multiple births (AOR 12.93 95% CI 5.45–30.72), rapid repeat childbirth (AOR 2.20 95% CI 1.34–3.63), and pregnancy termination (AOR 1.89 95% CI 1.11–3.23). With respect to social impacts, multivariable regression showed that girls married under age 18 remained at higher odds of engaging in age-disparate relationships (AOR 2.06; 95% CI 1.24–3.41) and of reporting FGM (AOR 2.14; 95% CI 1.11–4.13). In terms of maternal health outcomes, women married under age 18 were still significantly more likely to report receiving no ANC care (AOR 0.39; 95% CI 0.19–0.80), and less likely to deliver through C-section (AOR: 0.53; 95% CI 0.34–0.83). Institutional delivery and low birth weight were no longer associated with child marriage in the adjusted models.

We additionally assessed whether some of these associations were merely the result of the increased opportunity for reproduction that resulted from the fact that child marriages took place earlier than marriages above age 18 [5] by including duration of marriage in some of the final reproductive health models. After adjusting for marriage duration, the relationship between child marriage and ever use of contraception flipped, and women married under age 18 had lower odds of reporting ever using contraceptive methods (AOR 0.26 95% CI 0.14–0.47). Other outcomes such as rapid repeat childbirth, multiple births, and pregnancy termination were no longer associated with child marriage after adjusting for marriage duration.

### Qualitative findings

Analysis of qualitative study findings reinforced and expanded many of the findings generated through the quantitative analysis of DHS data. We classify findings pertaining to the impacts of child marriage into the following five themes which emerged as most salient: (1) Access to and use of sexual and reproductive health services; (2) exposure to FGM; (3) marriage and birth registration; (4) marital relations; (5) relationship with in-laws.

### Access to and use of sexual and reproductive health services

There was a widespread understanding among health providers interviewed in this study that child brides seldom came in contact with the health system until their first pregnancy. Several girls expressed their reluctance to seek public services because they did not have marriage certificates and many turned to private health providers instead. It was noted, however, that the cost of private health care was substantially higher than that of public services which was cited as a disincentive to seek care.

Gatekeepers were increasingly common in this setting and accompaniment by mothers and mothers-in-law was an expectation by providers interviewed. This was in spite of the absence of a legal minimum age at which girls can receive sexual and reproductive health services. The presence of a mother/mother-in-law, first degree relative, or husband was an implicit requirement by health providers particularly if a girl was seeking an “invasive” contraceptive method such as an intrauterine device or implant. Again, a husband’s or guardian’s consent for provision of family planning methods was not required by law, but their presence was considered an unspoken rule in most places.

As articulated by health providers:

“Look, if a girl is 15 years old, she needs to have someone from her relatives with her because 15 is a very young age so for me to be sure that this girl is actually married, she needs to have a family member with her. That’s more important than a national ID.” Health provider, Damietta

“If she is 15, she should not even seek an intrauterine device (IUD) insertion, she needs to have her mother or her husband with her before I can do this.” Health provider, Qalyubia

Several child brides interviewed in the study expressed fears around the use of family planning and were reluctant to use any form of contraception. However, others espoused favorable beliefs towards contraceptive methods, including long-acting ones. Mothers, on the other hand, were overwhelmingly opposed to their daughters using family planning methods before having their first child. Asked by an interviewer about what advice she would give to her daughter if she is seeking to delay pregnancy, a mother in Qalyubia remarked:

“I will tell her no. She must first have a child, then she can do whatever she wants. The important thing is for her to have a child first.” Mother, IDI, Qalyubia

Another mother in Damietta underscored the importance of having a baby immediately after marriage, stating that a “family is only complete after the birth of a baby.” Very prevalent among both young and older participants was the belief that having children early in marriage was a social imperative, enforced by husbands, mothers, and in-laws, and that failure to do so was not without consequence.

There were mixed views among health providers about the use of contraception among young girls. Some providers expressed their hesitation to insert IUDs for girls under age 18, but others actually preferred copper IUDs over hormonal methods which they said may cause infertility for young girls. The most appropriate methods were thought to be barrier methods like condoms. The following quotes demonstrate the diversity of opinions among health providers about contraceptive methods that are suitable for girls married under age 18.

“It is not advisable for a girl who has not given birth to insert an IUD. It is better for her to take the contraceptive pill because the IUD has several side effects for young girls and can cause bleeding. She is too young for it.” Health provider, Damietta.

“If she is young, an IUD is a preferred method. Hormones are not advisable, because of their implications for future pregnancies.” Health provider, KII, Giza

“For young girls, we do not recommend injectables because they can cause infertility.” Health provider, KII, Qalyubia

### FGM and child marriage

Interviews revealed an indirect link between FGM and child marriage; participants who espoused beliefs favoring FGM also tended to hold accepting views towards child marriage. Nonetheless, rarely did participants understand FGM as a direct pre-requisite for or immediate consequence of marriage. Few cases were cited in which a husband or suitor explicitly requested that a girl be cut as a condition for marriage. Only one case was cited in which a girl had been cut before her wedding day:

“I cut my daughter a few days before her wedding. Because of the groom’s mother. She asked that the girl be cut. Same thing happened with my aunt’s daughters. On the day of their wedding, their husband’s refused to consummate the marriage until the girls got cut. They were cut on their wedding day.” Mother, FGD, Qalyubia

It is worth noting however that many of the reasons proffered by participants for cutting or wishing to cut their girls were indirectly linked to marriage. For example, several participants stated that FGM was crucial for the preservation of girls’ purity and honor—which are ultimately tied to their marriageability. Others noted the importance of FGM for the beautification of girls’ genitalia—through the removal of excess skin—which eventually served to please a girl’s future husband.

“My older sister was cut, but we [in reference to her and her sister] still haven’t been. Our mother says that there is extra flesh that affects girls when they get married so they have to be removed.” Unmarried girl, IDI, Giza

“It has to be done to preserve values of chastity and honor in society. If we don’t do it, promiscuity will spread, especially that girls now have phones. When they are not cut, girls become promiscuous. Or so I have heard.” Father, FGD, Qalyubia

### Marriage and birth registration

Marriage registration emerged as a salient issue across interviews. Key informants explained that child marriage was banned in Egypt and that marriages under age 18 could not be registered. Most married girls interviewed in the study did not possess marriage certificates, and instead relied on temporary contracts which are not registered with the state or on *“wasl amana”* which is equivalent to a check that a girl can cash in the event of a divorce. Most participants noted that as soon as they turn 18, they will obtain a marriage certificate to ensure that their rights were protected. As recounted by one child bride in Giza:

“My aunt requested a “wasl amana” from my husband and took a copy of his national ID and birth certificate. In the beginning, the marriage registrar did not wish to marry us and asked my family how they were letting a girl marry so young, because I looked younger than I was, but then my mother and aunt convinced him that it was not his decision to make and he succumbed to them.” Married girl, FGD, Giza

In addition to lacking marriage certificates which underpin girls’ ability to claim many rights under Egyptian law, child brides were also unable to register the births of their children. In the absence of proof of marriage, birth registration is not legally possible in Egypt, and participants stated that they had to wait until their marriage was officiated before they could issue a birth certificate for their children. All children born in health facilities, however, were granted a birth notice, which was a document attesting to the birth of a child in a given hospital or health clinic. If a parent was unable to furnish a birth certificate, a birth notice was presented to health providers to obtain vaccinations and other health services for the child. There was consensus among the health providers interviewed that children would readily receive vaccinations in the absence of legal documents because children’s safety and survival came first. Nonetheless, a few participants reported facing difficulties vaccinating their children in the absence of legal documents that proved that the child in question was theirs. One married girl in Giza noted:

“I still have not been able to issue a birth certificate for my son. When it is time for his vaccination, they ask me in the health center for his birth certificate. They then interrogate me, asking for my national ID, how I got married etc. They make it really cumbersome. Usually, I give them my birth certificate and national ID, but sometimes they will say these are not proof that this kid is yours. We need to make sure you are his mother. I have a report from my doctor who delivered me, but they gave me a hard time before they accepted it.” Married girl, Giza, FGD

In a very few cases, participants recounted cases where the husband passed away or simply left before the marriage could be registered. This created a host of problems, because neither the marriage nor the births resulting from a marriage could be registered. In such cases, children would be registered using the maternal grandfather’s name, for example, seeing as a birth certificate could not be issued with only the mother’s name on it.

### Marital relations

Most married girls confirmed that there was an age difference between them and their husbands. Interestingly, both married and unmarried girls consistently expressed a preference for their husbands/future husbands to be 5–8 years older, but not more. Nonetheless, in their own marriages, they expressed feeling dismissed and sidelined in decision-making because of their young age and noted difficulties in communicating with their partners and understanding their needs. As articulated by different participants in one FGD in Giza:

“My husband treats me like a child. Even though he is not that old, he is only 26. Whenever we argue about anything, he tells me your brain is too small, you have the brain of a child.”

“Whenever I try to give him advice, he tells me to ‘sit aside, can’t you see the difference between you and me, do you expect that you will be able to provide me with advice?’”

“He does not understand me. Sometimes I want to communicate something with him and I just can’t seem to get him to understand.”

Many married girls admitted that they experienced physical violence from their spouses. For some, this violence stopped or abated with time. Indeed, accounts from mothers and key informants confirmed that violence early on in marriage was more common because girls needed to be “shaped” or “molded” by their husbands, and thus needed ‘disciplining’. This was again said to reduce with time as a girl got older. Most respondents recognized violence as problematic but did not think it warranted divorce. One girl in Giza was beaten while 4 months pregnant, but her family did not see it as reason for a divorce:

“I went to visit my aunt at her house. She is the one who had married me off to my husband. I was one hour late. When he came to pick me up, he was calm as if there was nothing wrong. My husband does drugs, so he was high. Everyone does drugs you know. He took the curtain rod and kept hitting me with it. He hit and hit and would not stop. I almost died. We live in an isolated place in Giza so no one could hear my screams. But eventually my neighbor broke into our apartment and rescued me from him. When I told my aunt, she said it was normal for a husband to hit his wife. I told her I agree but only if a woman does something wrong. I did nothing wrong. She told me to go back to your house and your husband.” Married girl Giza FGD

### Relationship with in-laws

Verbal and physical violence were not exclusively perpetrated by the husband, with many girls recounting stories of abuse from their mothers- and sisters-in-law. Many participants lived with extended families in which mothers in law took an active part in decision-making around the household, dictating girls’ schedules and deciding whether they can leave the house for social visits. The social expectation that a girl should serve her in-laws was pervasive, especially among girls who came from rural areas. Several girls reported being subjected to abuse by their in-laws if they made mistakes or were not obedient.

“In the beginning, she [in reference to the mother-in-law] used to like me. After I gave birth, her son started to stay with me more, so she and her daughters came to my apartment, and gave me a good beating.” Married girl, Giza, FGD.

“A mother-in-law is supposed to be like a mother, but she seldom is. My mother-in-law once took me to the street and beat me in front of everyone.” Married girl, Giza, FGD

## Discussion

To our knowledge, this is the first study that investigates the prevalence and consequences of child marriage in urban settings in Egypt, the MENA region’s most populous country [24]. Our findings indicate that child marriage is still occurring in urban areas and more than 1 in 10 women aged 20–24 are married prior to the age of 18. While lower than the overall national estimate (17.4%), prevalence in urban areas in Egypt is higher than that in other countries in North Africa such as Tunisia and Algeria, yet lower than countries like Morocco and Mauritania [25]. The prevalence of child marriage under the age of 15, on the other hand, was 2.5%, which is higher than the country’s national average of 2% but lower than that of the MENA region’s which is 3% [25].

Our quantitative analysis of consequences of child marriage complements the literature on consequences of child marriage. Like other studies, we find that child marriage increased the risk of poor fertility outcomes such as multiple births, rapid repeat childbirth, and pregnancy termination, all of which persisted after adjusting for sociodemographic characteristics [5, 15, 26–30]. That these associations disappeared after adjusting for marriage duration could mean that the longer duration of child marriages which prolongs the exposure risk to multiple and rapid repeat pregnancies in the reproductive life span is what drives the high fertility and poor fertility outcomes observed among girls married under age 18. Unlike some studies, however, we did not find evidence that child marriage was associated with childbirth in the first year of marriage or unwanted pregnancy. As pointed by Gage et al., potential explanations for the lack of a significant relationship between child marriage and childbirth in the first year are the “low fecundity at very young ages, low coital frequency, or both.” [30] The absence of an association between unwanted pregnancy and child marriage could stem from strong pronatalist norms in this setting, which encourage childbearing.

We found that girls married as children are increasingly less likely to ever use contraception compared to their counterparts who are married as adults, after adjusting for sociodemographic covariates and marriage duration. This finding resonates with our qualitative analysis which revealed that a subset of girls were reluctant to use contraceptive methods due to negative attitudes and misconceptions about them, which were shared by mothers and reiterated by some health providers. Our finding that gatekeeping is common and that accompaniment to care by mothers, mothers-in-law or husbands is an implicit requirement in some healthcare settings may be in part responsible for the increased risk of never use of contraception in this group. Indeed, evidence suggests that requiring parental or spousal consent can act as a strong barrier for girls and women to access sexual and reproductive health services [31–33]. In Egypt, despite progressive standards and guidelines that enforce reproductive rights and freedoms, enforcement is inconsistent and denying women services—while not allowed by law—still occurs [33].

Potentially compounding this is the low support of health providers for the provision of certain contraceptive methods to adolescent girls, an issue which warrants attention and policy intervention. Even if provider preferences do not lead them to deny services to young girls, negative attitudes and biases can push girls towards specific methods and compromise their ability to make informed choices. Educating health providers about the safety of contraceptive methods for adolescent girls can thus ensure that girls have a wide method mix from which to choose and sound information upon which they can base their decisions. This finding is not unique to Egypt, and several studies have documented restrictions on services by providers based on marital status, age and birth history [32–34]. How often these restrictions are actively imposed, which contraceptive methods they impact the most, and how they ultimately impact contraceptive uptake in this setting are areas requiring further research.

Our quantitative analysis also sheds light on several social outcomes of child marriage. Child brides were more likely than women married as adults to be in marriages characterized by large spousal age gaps. This is consistent with various studies on child marriage which confirm that large spousal age gaps and ensuing power imbalances characterize these marriages [6, 7, 37]. Large spousal age gaps can diminish from girls’ autonomy and decision-making and predispose them to intimate partner violence [7]. Our qualitative data illustrates how large age gaps which were very common in our qualitative study sample led girls to feel they could not communicate with their partners or voice their opinions. Several girls also admitted to experiencing verbal abuse and physical violence from their husbands which several participants attributed to a process of “molding” that takes place in the early stages of marriage. The large spousal age gap that characterize these relationships in turn “facilitate this ‘character molding’ of younger brides.” [6].

We also found evidence of a strong statistical association between female genital mutilation and child marriage in this setting. While our qualitative findings did not provide much evidence of a direct link between FGM and child marriage (rarely was FGM considered a precondition for child marriage), they did point to an indirect link between the two practices. Both FGM and child marriage are borne out of deep-seated fears around girls’ engagement in premarital sex and a strong concern with girls’ virginity and family honor [14, 38, 39]. Both practices signal attempts to control female sexuality and are sustained and perpetuated by similar social and gender norms. It is thus perhaps not surprising that quantitative analysis revealed that girls who married early were also more likely to experience cutting compared to their counterparts who married at a later age.

Our quantitative analysis additionally uncovered associations between child marriage and a set of maternal health outcomes. However, many of these associations disappeared after accounting for the effects of wealth and education, which could indicate that child marriage is linked to poor maternal health outcomes through its association with socioeconomic status. For antenatal care and delivery by C-section, adjusting for socio-demographics attenuated these relationships but did not eliminate them entirely, indicating that poverty may not be the sole pathway through which child marriage affects these specific outcomes. That child brides were less likely to seek ANC and less likely to undergo C-section deliveries (although still high) is in line with studies conducted in various settings that find that child brides are less likely to seek healthcare generally, including prenatal and postnatal care, and are less likely to deliver in facilities [40–45]. Reasons proposed for these associations are the limited power and decision-making of child brides in negotiating health care utilization as well as low access to resources [30]. Qualitative findings help contextualize this finding by drawing attention to the reluctance of some participants to seek health services in the absence of marriage certificates. This has led some participants to prefer to go to private providers who often charge high user fees, thus potentially disincentivizing care seeking.

Qualitative analysis also shone a spotlight on the issue of marriage and birth registration. The current legal system prevents registration of marriages below the age of 18 to discourage child marriage. However, one dangerous implication of this practice is that children born to child brides cannot be registered without the presence of a marriage certificate. This can curtail or delay children’s access to important services and can eventually increase their own risk of child marriage if they are unable to prove their age [46]. While it was evident that birth registration eventually takes place after a girl turns 18, delays in registration are not without problems and if for any reason, a father is no longer present, birth registration is not permitted in the absence of a father. While a birth notice can be used in lieu of a birth certificate to enable a child to receive most—albeit not all—services, children not born in health facilities are unable to obtain this notice, impacting their ability to access important services. There is thus need for concerted advocacy efforts to identify an acceptable legal solution that allows birth registration irrespective of marriage status so that children can be immediately registered, and their legal identity can be established, allowing the preservation of their rights and entitlement to services.

Quantitative study findings should be considered in light of some limitation. First, we were constrained by the lack of more updated nationally representative data and were forced to rely on DHS 2014 which may not fully reflect current practices related to child marriage. Further, the sample of women aged 20–24 may provide a “lagged” picture of consequences of child marriage because women were married as children at least three years prior to the time of the survey.^2^ In contrast, the qualitative sample comprises girls aged 10–19 and therefore provides a more current picture of the experience of child brides. Because of the difference in age groups used and timing of data collection, the two samples are not comparable especially in light of sociopolitical and legal changes that may have occurred in the time separating the two age cohorts. Nonetheless, we believe that the health and social outcomes examined in this study were likely unaffected by political and social events that may have taken place, and therefore, the analysis still sheds light on the consequences of child marriage on women and girls. Another limitation pertains to the use of cross-sectional data which limits our ability to draw causal inference and understand temporality. We also relied on self-reported measures which are subject to recall and social desirability bias. It is possible that respondents misrepresented their age and age at marriage, or inaccurately reported information relevant to their fertility due to stigma or social taboos. They may have also poorly recalled certain behaviors such as healthcare utilization. Third, we were limited by the sample size of girls aged 20–24 in EDHS, so some of our confidence intervals were wide. Additionally, it would have been more relevant to “lag” some of our outcome variables to capture practices occurring in early stages of marriage rather than the present; however, information reflecting practices, such as contraceptive use or pregnancy termination in the first year of marriage, were unavailable. Our qualitative analysis also has some limitations. First, we relied on existing data that was collected as part of another study investigating child marriage practices among Syrian refugees and Egyptians residing in urban areas in the three study sites. However, that study yielded rich data capturing the experiences of Egyptians residing in these sites. Second, the data was elicited from participants residing in three governorates only and thus the extent to which findings are transferable to a broader population is uncertain. Lastly, participants may have misreported attitudes or behaviors in the presence of the interviewers, especially when discussing sensitive issues. However, the presence of well-trained and seasoned data collectors with ample experience conducting research in Egypt may have attenuated this type of bias.

## Conclusion

Our study provides important insights about child marriage in urban areas in Egypt and illustrates a range of negative outcomes associated with the practice. While identifying effective interventions that can address the challenges faced by child brides is outside the scope of this study, our findings underscore the importance of interventions that delay the initiation of marriage and reproduction as well as policies and programs that recognize the unique risks and acute vulnerabilities faced by married girls.

## Data Availability

The qualitative dataset supporting the conclusions of this article is available on request. The 2014 EDHS questionnaire and dataset are available through the DHS website and data repository which can be accessed using the following link: https://dhsprogram.com/methodology/survey-search.cfm?sendsearch=1&str1=10,&crt=1&listgrp=0.

## Abbreviations

AOR: Adjusted odds ratio
ANC: Antenatal care
CI: Confidence intervals
DHS: Demographic and health survey
FGM: Female genital mutilation
FGDs: Focus group discussions
IDIs: In-depth interviews
IRB: Institutional Review Board
KIIs: Key informant interviews
MENA: Middle East and North Africa
MOHP: Ministry of Health and Population
NPC: National Population Council
OR: Odds ratios
PSU: Primary sampling unit

## Footnotes

1 Restricting the quantitative analysis to women ages 20–24 ensures that sufficient time had elapsed for respondents to experience the outcomes of interest while providing recent rates of child marriage prevalence. This age range is commonly used [5, 15, 16] and is consistent with the Sustainable Developments Goal indicator 5.3.1 [17].

2 A 20 year old woman who was married as a child, would have been married three or more years prior to the survey.
